# Suggestions for a sustainable medical quality-centered approach within a predictive, preventive and personalized medicine: the promotion of alternatives to conventional hospitalization in an era of financial constraint

**DOI:** 10.1186/1878-5085-5-S1-A156

**Published:** 2014-02-11

**Authors:** Guglielmo M Trovato

**Affiliations:** 1 Dipartimento di Scienze Mediche e Pediatriche – Unità di Terapia e Diagnostica Medica non Invasiva, AOU Policlinico-VE, University of Catania, Italy

## 

Alternatives to traditional hospital acute care for medical disorders address to benefits for the Health Systems, which experience budget constraints [1]. The comprehensive approach to the use of high-tech medicine for improved health care provided by the European Association for Predictive, Preventive, and Personalized Medicine (EPMA) is exactly along this line of intervention [2]. The rubber hits the road in the doctor-patient relationship, and the collective health-care knowledge must be applied to the particular patient's health-care situation [2]. A sustainable answers are the “Quick diagnosis units” [3] often reported with limited clinical description. In our Institution we shifted, beginning in 2004, part of the patients addressed by our emergency department toward an autonomous out-patient Diagnostic and Therapy Medical Unit (DTU), which has essential point of care laboratory test facility and certified competence of the Medical staff (two senior and two Junior Medical Doctors) in Medical Ultrasound, Echocardiography and dietary/physical exercise assessment and prescription[4], helped by a part-time dietitian, health psychologist and a nurse with intensive care experience. All procedures, including echo-guided diagnostic-therapeutic intervention, are performed by the components of the staff. Admittances are primarily oriented toward the care of patients with chronic liver disease, secondary anemia, severe malnutrition (including mental anorexia), heart failure; 25% of patients had a diagnosis of Cancer; 18% of patients were diagnosed by biopsies of various organs. The stay in DH was 6.3±2.1 non consecutive days, with a shortage of costs, with a case mix index (1.39±0.23. The DTU is a place of training for Post-Graduate MDs of the School of Internal Medicine and of the Postgraduate School of Medical Ultrasound. It provides quick and timely diagnosis, by ultrasound and lifestyle assessment, and articulated therapeutic strategies, including dietary and physical exercise prescription [5]. The critical contribution of DTU [3] is medical-quality centered more than medical-accountancy-centered. Greater focus on staff professional competences in sustainable diagnostic procedures, such as ultrasound, lifestyle assessment and dietary and health-psychology intervention is needed for enhancing effectiveness. Such strategy will fasten diagnosis and follow-up, allowing affordable therapeutic personalized approaches [4,5]. These issues were and are object of controlled studies that were and will be published on peer-reviewed high impact medical journals.

## Recommendations

PPPM experience and proposed models need to be monitored and assessed not only on the basis of financial sustainability and patient’s satisfaction, as currently is done by public and private institutions, but also by carefully designed research projects. These should be independently supported (such as EU funding). These must have the goal of overcoming the gap among predictive, preventive and personalized medicine mainly within a comprehensive urban daily life scenario. Calls must encompass the actual pre-requisite and promotion of the professional quality of staff skilled 1) in sustainable non-invasive diagnostic procedures, 2) in non-pharmacological intervention (diet, lifestyle changes), 3) in translational research(from epidemiology to personalized therapy) and 4) in timely dissemination of the most high impact information. An effective training of professionals toward these PPPM directives and planned articulated collaborations with of Academy, Health Institutions, Cities, non-profit organization and SMEs should be included in any project.
